# Dual-Channel Spectral Domain Optical Coherence Tomography Based on a Single Spectrometer Using Compressive Sensing

**DOI:** 10.3390/s19184006

**Published:** 2019-09-16

**Authors:** Luying Yi, Liqun Sun, Mingli Zou, Bo Hou

**Affiliations:** State Key Laboratory of Precision Measurement Technology and Instruments, Department of Precision Instrument, Tsinghua University, Beijing 100084, China; yily15@mails.tsinghua.edu.cn (L.Y.); houb15@mails.tsinghua.edu.cn (B.H.)

**Keywords:** optical coherence tomography, dual-channel image, imaging depth, imaging speed, compressive sensing

## Abstract

Dual-channel spectral domain optical coherence tomography (SD-OCT) is one of the effective methods for improving imaging depth and imaging speed. In this paper, we design a dual-channel SD-OCT system based on a single spectrometer that can operate in two modes: (1) Increasing imaging speed and (2) expanding imaging depth. An optical path offset is preintroduced between the two channels to separate the two-channel data. However, this offset increases the requirement for the spectral resolution of the spectrometer in mode (1), so compressive sensing (CS) technology is used herein to overcome this problem. Consequently, in mode (1), when the spectral resolution of the spectrometer is the same as that used in the single-channel system, we use a dual-channel SD-OCT system combined with CS technology to double the imaging speed. In mode (2), when the spectral resolution of the spectrometer is only half of that used in a single-channel system, the imaging depth can be nearly doubled. We demonstrate the feasibility and effectiveness of the method proposed in this work by imaging a mirror, a fish fin, a fish eye, and an onion.

## 1. Introduction

Spectral domain optical coherence tomography (SD-OCT) as one kind of OCT has become a very important biological imaging modality, especially in ophthalmology, because of its advantages of high resolution, fast imaging speed, and high signal-to-noise ratio (SNR) [1,2,3]. Imaging speed and imaging depth are two significant performance indications for common imaging technologies. In SD-OCT, the imaging speed depends mainly on the scanning speed of the sample arm and the acquisition speed of the spectrometer. The imaging depth depends mainly on the penetration depth of the incident beam in the sample and the spectral resolution of the spectrometer. Multi-channel OCT systems [4,5,6] and optical computing [7] have been shown to effectively improve imaging speed. Different approaches have been taken to increase the imaging depth, such as multi-channel OCT systems [4,5,6], interferometric synthetic aperture microscopy (ISAM) method [8], Bessel beams OCT systems [6,9], etc. Accordingly, multi-channel SD-OCT is one of the effective methods for simultaneously improving imaging depth and imaging speed. When the scanning speed of the scanning galvanometer is constant, the multi-channel SD-OCT system can shorten the imaging time and it can enlarge the imaging depth when the light beams of different channels are focused at different depths of the tissue. Although the imaging depth can be increased by increasing the depth of focus (DOF) of the beam, there is a trade-off between the lateral resolution and DOF in SD-OCT based on Gaussian beam. Therefore, stitching several beams with a small DOF into one beam can obtain higher lateral resolution and greater SNR [5,6,10].

In multi-channel SD-OCT, the separation of spectral data from different channels is very significant. Most of the existing multi-channel SD-OCT systems are implemented by multiple spectrometers or fast optical switches [11,12,13,14], but these methods increase the cost and complexity of the system or increase the imaging time. Therefore, the SD-OCT system based on a single spectrometer has become a hot research topic. It has been demonstrated that introducing optical path offsets between different channels in advance is an effective way to separate spectral data from different channels [15,16,17,18,19,20]. This method was originally developed for polarization-sensitive SD-OCT systems based on a single spectrometer to separate images of two polarization states [15]. Later, a bidirectional SD-OCT system based on a single spectrometer to enhance the imaging depth [19] and a dual-beamlet phase-sensitive SD-OCT for measuring vibration were reported [16]. Although they all prove that this method of introducing offsets is effective in SD-OCT, the offsets will lead to a reduction in imaging depth when the spectral resolution of spectrometer is certain. This is because the spectral resolution of the spectrometer determines the maximum optical path that can be detected, and the introduction of the offsets results in a larger optical path, thus requiring a spectrometer with a higher spectral resolution.

To solve this problem, we propose to use the undersampling method to reduce the high spectral resolution requirements of the spectrometer. Several algorithms have been proposed to reconstruct undersampled data, such as neural networks (NNs) reconstruction algorithms, compressive sensing (CS) reconstruction algorithms, etc. The neural networks algorithm has been widely used to reconstruct undersampled data [21,22], but it requires the input of a large number of images in advance to train the networks. CS-OCT technology has emerged as an increasingly popular research topic, with a view to reducing the imaging time. It has been demonstrated that high-quality OCT images can be reconstructed using approximately 30% spectral data and CS reconstruction algorithms [23,24,25,26,27,28]. Both the pretraining of deep learning and the subsequent processing algorithms of CS are time-consuming. In this paper, the CS algorithm is used to reconstruct the undersampled data. This is because the conjugate image is removed in this work by a dispersion encoding (DE) method that requires iteration [23,29,30], so it can be combined with the CS iterative algorithm. The method of combining DE and CS was described in our published work [23], in which full depth images can be reconstructed from undersampled data by only a few iterations, thereby greatly reducing processing time.

In this study, we design a dual-channel SD-OCT system based on a single spectrometer. Dual-channel spectral data are separated by introducing an optical path offset between two channels in advance, and the CS technology is used to reduce the high requirement of spectral resolution of spectrometer. We use this dual-channel SD-OCT system in combination with CS method to double the imaging depth (in mode 2) and imaging speed (in mode 1) without increasing the spectral resolution of the spectrometer.

## 2. Methods

### 2.1. Dual-Channel SD-OCT

**Figure 1:**
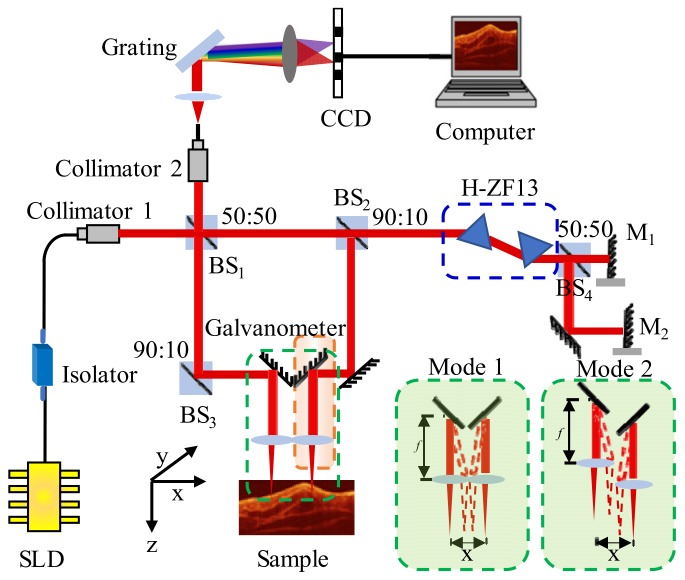
Experimental setup for dual-channel spectral domain optical coherence tomography (SD-OCT). BS: unpolarized beam splitter; M: mirror; the diagram within the green dashed box is a schematic of two imaging modes, in which *f* is the focal length of the focusing lens.

A 790 nm dual-channel SD-OCT experiment setup is shown in Figure 1. The light with 40 nm bandwidth emitted from a superluminescent diode (SLD-331, Superlum Ltd, Russia) is transformed into collimating spatial light through the optical fiber collimator 1. After the reference light is split by BS_1_ and BS_2_, the dispersion is introduced into reference light by a pair of dispersion prisms (H-ZF13). Then, it is divided into two equal light beams through BS_4_, which are reference lights of channel 1 and channel 2, respectively. The sample light of channel 1 reaches the sample after being split by BS_1_ and BS_3_. The sample light of channel 2 is split by BS_1_ and BS_2_, and then arrives at the sample. In the sample arm of channel 1, BS_3_ is used instead of a mirror in order to make the intensity of the sample light in both channels consistent, and both beams pass through the two beam splitting prisms to reach the sample. The sample lights and reference lights interfere at BS_1_ and are then incident into collimator 2, which are finally collected by a spectrometer (Maya 2000 Pro, Ocean Optics) with a spectral resolution of 0.05 nm. The axial resolution in air is approximately 6.88 μm. The focal length *f* of the focused lens used in mode 1 is 30 mm; and that used in mode 2 is 15 mm. Further details about the system configuration can be found in Table 1.

The sensitivity of the proposed dual-channel SD-OCT configuration was calculated and measured. A neutral-density filter followed by a mirror was used to create a sample with a reflectivity of −56 dB. The light power incident on the sample was about 1.3 mW, and the integral time of the spectrometer was set to 8 ms. The charge-coupled device (CCD) has a quantum efficiency of 80% at 800 nm, a dark current of 50 e-/pixel/s (25 °C), and a readout noise of 6 e- rms. The SNR, in dB, was calculated as twenty times the base-10 logarithm of the ratio of the A-scan peak height to the standard deviation of the noise floor. The noise floor standard deviation was taken at the location of the A-scan peak by blocking the sample arm. The calculated sensitivity was 111 dB [2,31], and the measured sensitivity of each channel was about 103 dB. The difference between the two values may be due to the low efficiency of the spectrometer, the residual DC signals and phase fluctuations, etc [2]. It should be noted that since the reflectivity of the sample arm is usually much smaller than the reference arm, the power used for imaging in the system was increased as much as possible, but we have not optimized the reference arm power to optimize the system sensitivity [2]. The CCD of the spectrometer used in the system had a full well capacity (FWC) of 200ke- and the number of pixels used was 960. The two reference beams share the same FWC of the CCD, so the theoretically estimated dynamic range of each channel was not more than 70 dB [3,32].

In each channel of the SD-OCT system, the galvanometer scanner and focusing lens are connected together by a lens barrel and mounted on a 3D motorized stage for lateral and axial alignment of the system. The y-direction stage is used to align the sample arm scanning beam such that the scanning points of the two sample arms are on the same line parallel to the x-axis. The z-direction stage is used to adjust the focus position of the two sample beams.

**Figure 2:**
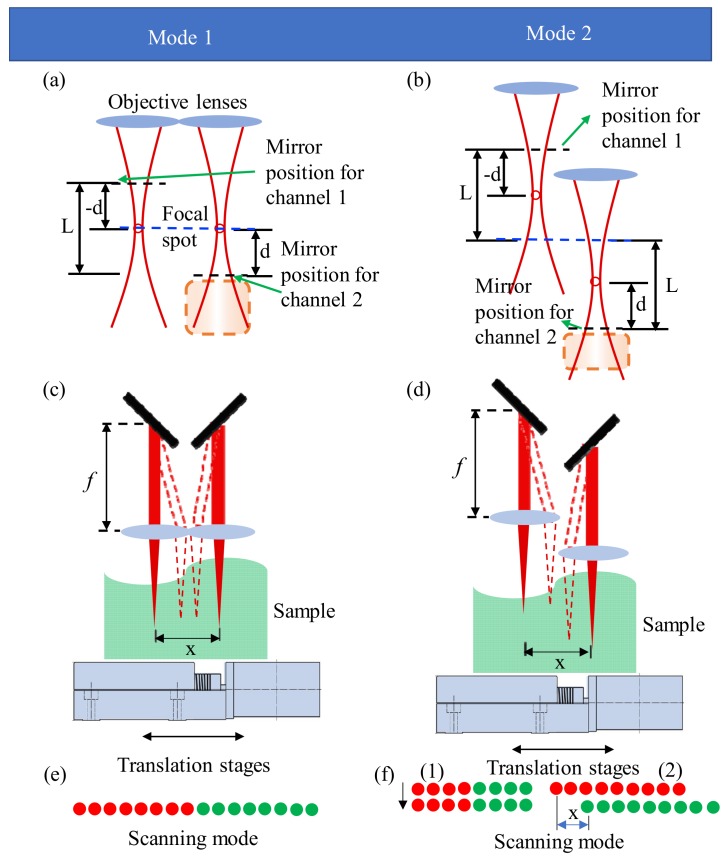
(**a**,**b**) show position relationships between the focused sample beams and the reference mirrors in mode 1 and mode 2, respectively; (**c**,**d**) are schematic diagrams of sample arms in the experiment setup; (**e**,**f**) are scanning modes of two imaging modalities. In (**a**,**b**), the blue dotted lines represent the positions of coordinate origin along the depth *z* direction, and the black dotted lines represent the positions of the zero optical path difference of each channel.

Figure 2 is the schematic diagram of effective position relationship between the focused sample beams and the reference mirrors in each imaging mode. Figure 2a shows the position relationship between the two channels in imaging mode 1, and the intensity of backscattered signal from the out-of-focus scatters within the red dotted box are substantially suppressed by the confocal gate [33]. We assume the coordinate origin along the depth direction is at the focal spot for simplicity, and then the optical fields of the two reference lights can be expressed as [15]

(1) $$\begin{array}{l} E_{1}^{r}=R\exp[i2kd]\exp(i\varphi (k))\\ E_{2}^{r}=R\exp[-i2kd]\exp(i\varphi (k)) \end{array}$$

where *R* is the reflectance of reference arm, *k* is the wave number, and phase φ(k) is introduced due to dispersion in order to remove conjugated image [23,30]. The signals of two sample arms are

(2) $$\begin{array}{l} E_{1}^{s}=\int S_{1}(z)\exp[i2kz]dz\\ E_{2}^{s}=\int S_{2}(z)\exp[i2kz]dz \end{array}$$

in which *S*(z) is the amplitude of backscattered light field at a depth of *z* in sample. The interference spectrum collected by spectrometer is

(3) I(k)=2Re{R[exp(iφ(k))∫−ddS1(z)exp(i2k(z+d))dz… +∫−ddS2(z)exp(i2k(z−d))dz]}

It can be seen that the signals of two channels are spatially separated by 2*d*, and the optical path range of the sample to be imaged is [0, 2*d*] in channel 1; while that is [−2*d*, 0] in channel 2. Consequently, there is no overlap between the two channels when the conjugated image is removed. However, this method sacrifices half of the imaging depth, as shown in Figure 2a. For example, if the spectral resolution of spectrometer can satisfy the obtaining imaging depth of *d*, i.e., *L*/2, we can get the imaging depth of *L* in the conventional single-channel SD-OCT system after removing the conjugated image. However, the spectrometer is required to measure the imaging depth of *L* to obtain the imaging depth of *L* in the dual-channel SD-OCT system. In order to reduce the high requirement for spectral resolution of spectrometer, we randomly read the spectral data of the spectrometer that can measure the depth of *d*, then reconstruct the signals of *L* depth using the CS algorithm.

Figure 2b shows the position relationship between two channels in imaging mode 2, in which the theoretical imaging depth is 2*L*. In a traditional single-channel system, the spectrometer needs to be able to measure the depth of *L* to obtain 2*L* imaging depth. In a dual-channel system, even without using CS technology, the spectrometer also needs to measure the depth of *L* to obtain the imaging depth of 2*L*. However, there is no doubt that using the CS technology can theoretically reduce the requirement for spectral resolution of the spectrometer.

Figure 2c,d are setup schematic diagrams of the sample arms in two modes, in which the rotating axis of the scanning mirror is placed at the focal point of the lens, so that the total scanning range *x* can be adjusted according to actual application. Figure 2e,f show the scanning modes in two imaging modalities. The red and green points in Figure 2e,f represent the scanning ranges of channel 1 and channel 2, respectively. The two channels are simultaneously scanned in mode 1, so the imaging time is reduced by half. There are two scanning schemes for mode 2. The first way is to scan the sample with the galvo scanner, as shown in Figure 2f-1. The upper structure of the sample is first scanned, and then the fast z-direction lifting table is used to change the focus position of the two sample beams to scan the deeper structure of the sample. The second way is to scan the sample by moving the sample arm or sample, as shown in Figure 2f-2. The upper and deeper structures of the sample are scanned simultaneously by the sample scanning beams of channel 1 and channel 2, respectively, then the lateral misalignment present in structural image is corrected. In mode 2, the imaging time is the same as that in the single-channel system when their scanning ranges are the same, but the imaging depth is doubled in the dual-channel system.

However, it has to be pointed out that there are two disadvantages in this dual-channel SD-OCT system. First, in order to simultaneously achieve two imaging modes and form a telecentric optical path, each sample arm has a separate focusing lens, which results in a scanning dead zone between the two channels. To solve this problem, a fast x-direction motorized stage with high-positioning accuracy needs to be moved once to compensate for the dead zone. This process takes approximately 10 milliseconds, which is much less than the total imaging time, or even less than the time required for one depth scan. When the imaging range in x direction is small, it can be scanned by moving the sample or sample arm using the fast x-direction motorized stage. In this case, there is no such dead zone, but the imaging time and the applicable fields are limited.

Secondly, in Figure 2a,b, the backscattered signal within the red dashed box away from the DOF is considered so weak that its interference with the signal of channel 1 can be ignored. For most highly scattering biological tissues, the OCT signals would rapidly fall into the noise floor for the optical depth beyond 2.0 mm [15], so this assumption is reasonable. For samples with a thickness less than *L*, there is no crosstalk between the images of the two channels, as shown in Figure 2a. However, for a relatively transparent thick sample, we need to reasonably set *L* according to the distribution of the backscattered signal of the sample, so that the backscattered signal within the red dashed box is weak.

### 2.2. Compressive Sensing SD-OCT

Equation (3) can be formulated as a digital signal as follows [23]:

(4) f=2RRe[ΦΨ(Sz1+d+Sz2−d)],

where $S\in C^{N}$ represents the full-range spatial signals; *z*_1_ and *z*_2_ are depth coordinates of the two channels, both of which are in the range of [−*d*, *d*]; $\Psi \in C^{N\times N}$ represents the discrete Fourier transform; and $\Phi =diag(\exp(i\varphi ))\in C^{N\times N}$, which represents the dispersive measurement matrix. We define the random sampling matrix as $T\in C^{M\times N}$, and the sampling rate is *M*/*N*. The vectors **S**_z1+*d*_ and **S**_z2-*d*_ can be reconstructed from a subvector **f**_u_ expressed as

(5) fu=2RRe[TΦΨ(Sz1+d+Sz2−d)].

CS recovers **S** by solving the constrained optimization problem, and the two-step compressive dispersion encoding (TCDE) method is used in this paper, which is introduced in detail in our previous work [23]. In the TCDE method, a large dispersion mismatch is introduced between the reference arm and sample arm to achieve strong suppression of mirror image artifacts, and two iterations are performed to reconstruct the full-depth OCT image from 1D undersampled spectral data. The first iteration selects the signal peaks with higher intensity and the signals in the previous and next pixels of these peaks, followed by the removal of their conjugate items and incoherent aliasing artifacts caused by undersampling. The second iteration selects the signals with lower intensity. If the structure of the tissue is complex, it is necessary to increase the number of iterations as appropriate.

**Figure 3:**
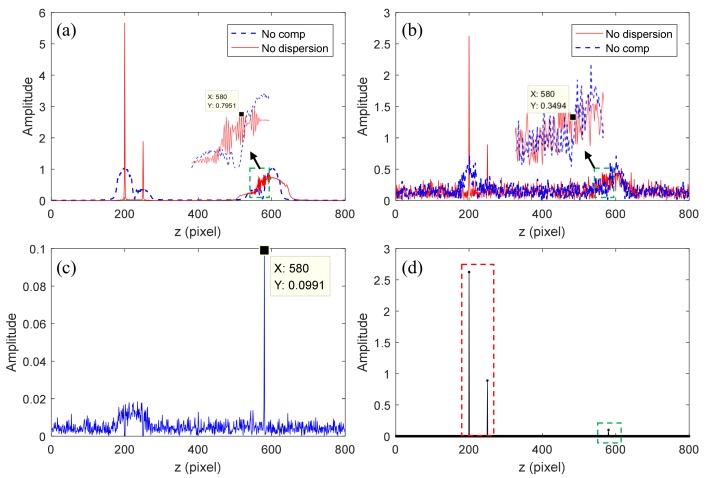
Simulation results of the two-step compressive dispersion encoding (TCDE) method. The results in (**a**,**b**) are reconstructed structural signals using 100% spectral data and 50% spectral data, respectively. The result in (**c**) is the residual signal with larger signals removed. The signals in (**d**) are the final reconstructed signals.

A simple simulation is conducted herein to illustrate the principles and steps of the TCDE method, and the results are shown in Figure 3.

The simulation conditions are the same as those used in Reference [21]. In the simulations, the spatial sparse signal is ***a***, where ***a*** (200) = 3, ***a*** (250) = 1, and ***a*** (580) = 0.1, and the other positions are 0. The results in Figure 3a,b are structural signals reconstructed from the fully sampled interferogram and the undersampled interferogram with a sampling rate of 50%, respectively. In Figure 3a,b, the blue lines represent the structural signals that have broadened because of uncompensated dispersion, and the red lines are the signals that have been compensated for dispersion, which implies that the real structure signals are enhanced and not broadened. By contrast, the conjugate signals are broadened and suppressed. We can find that small signal ***a*** (580) is submerged and cannot be extracted directly because of the interference peaks of large signals and the incoherent aliasing artifacts caused by random sampling. Hence, TCDE method is used to reconstruct the structural signal more accurately. In the first iteration, the larger signals shown in the red dashed box of Figure 3d are extracted and their conjugate signals and aliasing artifacts are removed. The residual signal is shown in Figure 3c, where only the small signal as well as its conjugate terms and artifacts remain. In the second iteration, the small signal shown in the green dotted box of Figure 3d is recovered. After two iterations, all structure signals are reconstructed, and their conjugate items are removed.

## 3. Results

**Figure 4:**
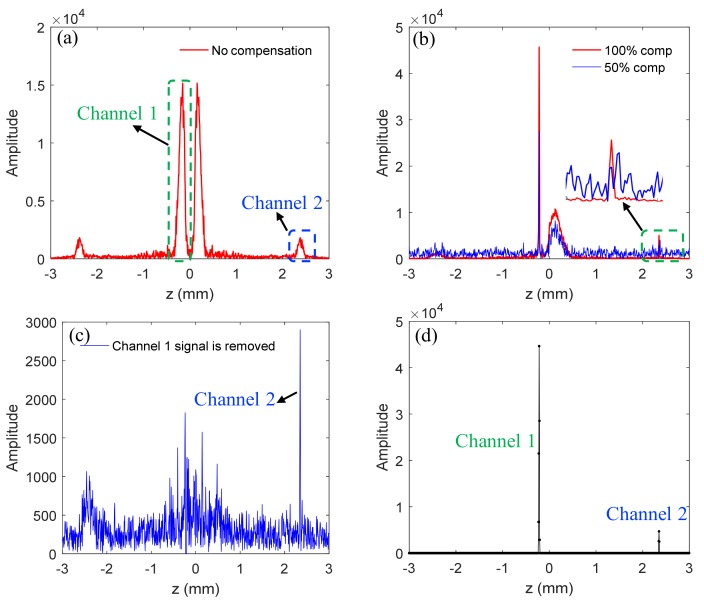
Experiment results of dual-channel SD-OCT system whose sample is a mirror. The red curve in (**a**) is the signal with dispersion. The red and blue lines in (**b**) are the structural signals after numerical dispersion compensation, which are obtained by 100% spectral data and 50% spectral data, respectively. The result in (**c**) is the residual signal with channel 1’s signal removed. The signals in (**d**) are the final reconstructed signals.

The feasibility of the method of combining dual-channel SD-OCT with CS is demonstrated based on the results for a mirror, as shown in Figure 4. The dispersion compensation coefficients are obtained [30]: $a_{2}=7680\times 10^{-30},a_{3}=376\times 10^{-45}$.

The red curve in Figure 4a is the structural signal that has broadened due to uncompensated dispersion, and the position difference of reference mirrors between channel 1 and channel 2 is 2.8 mm. It should be noted that because the sample mirror in channel 2 is farther away from the position of zero optical path, the signal intensity is lower compared to that in channel 1. In practical applications, the sample mirror should be set at the position of *z* = 0 to make the amplitude of the two channels’ signals the same. In this experiment, we deliberately set the sample mirror in another location to illustrate the relationship between the optical path and the sensitivity of the spectrometer. The red and blue lines in Figure 4b are signals that have been compensated for dispersion, which are obtained from 100% spectral data and 50% spectral data, respectively, from which we can see that the real structural signals are enhanced and not broadened, but conjugated signals are suppressed and broadened. Moreover, the signal of channel 2 is submerged in incoherent aliasing artifacts due to random undersampling. Therefore, we use the TCDE method to reconstruct the structural signals. After the first iteration, the large signal of channel 1 is reconstructed. Figure 4c shows the residual signal with the large signal as well as its conjugate items and aliasing artifacts removed. As can be seen from Figure 4c, only the small signal as well as its conjugate term and artifacts remain. Similarly, after implementing the second iteration, the small signal of channel 2 is recovered.

The results in Figure 4 show that the structural signal can be reconstructed from 50% undersampled data, indicating that the dual-channel SD-OCT combined with CS can double the imaging speed or imaging depth without increasing the spectral resolution of the spectrometer. Next, we explore the lowest sampling rate that this method can tolerate.

**Figure 5:**
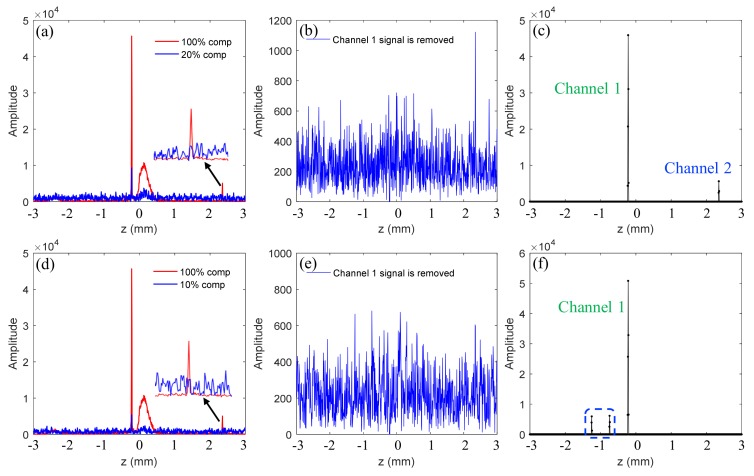
Experiment results of dual-channel SD-OCT system whose sample is a mirror. (**a**)–(**c**) The results are obtained by 20% spectral data; (**d**)–(**f**) the results are obtained by 10% spectral data.

Figure 5a–c are obtained using 20% spectral data, the contents of which correspond to what are represented by Figure 4b–d, respectively. The blue curve in Figure 5a shows that the signals are further compressed, and the small signal is still submerged in the incoherent aliasing artifacts and noise. After the larger signal is extracted and removed, the small signal can be extracted, as shown in Figure 5b. Finally, the two-channel signals are correctly reconstructed with 20% data. Figure 5d–f are obtained with 10% spectral data. In Figure 5e,f since the sampling rate is too low, the small signal is still submerged after the removal of the large signal and eventually cannot be recovered correctly. It has been demonstrated that when the sampling rate is 5%, the large signal cannot be reconstructed correctly all the time. Therefore, in the actual experiment, we should consider the sensitivity of the spectrometer to reasonably adjust the position of the scanning beam and the optical path of the sample depth, so as to make the signals of both channels as large as possible.

**Figure 6:**
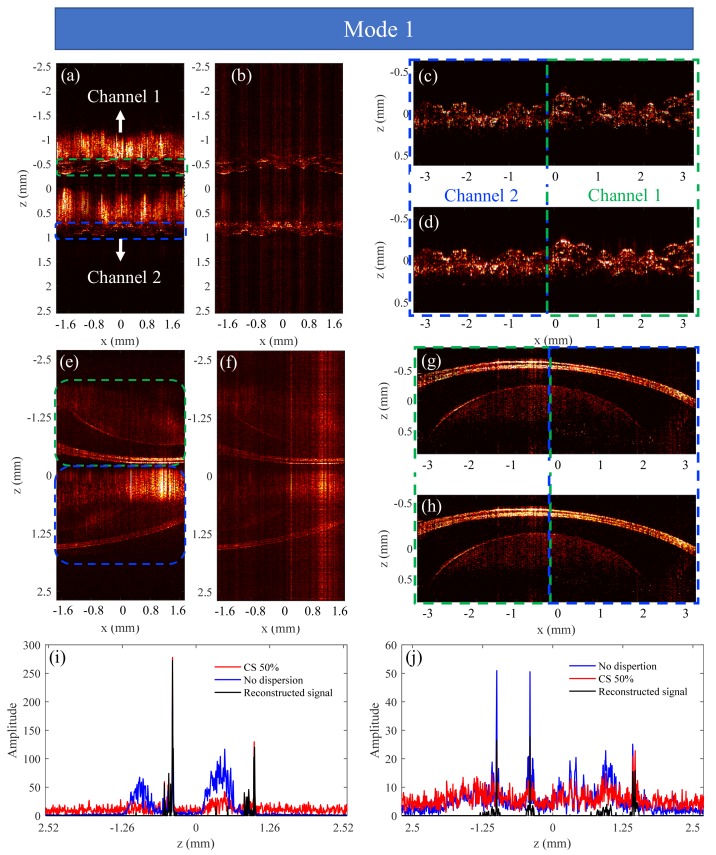
Experiment results of a fish fin and a fish eye. The results in (**a**,**e**) are obtained using complete spectral data, and the results in (**b**,**f**) are obtained using 50% spectral data. Panels (**c**) (50% data) and (**d**) (100% data) show images of a fish fin that are stitched together by the results of the two channels; Panels (**g**) (50% data) and (**h**) (100% data) show images of a fish eye that are stitched together by the results of the two channels; (**i**,**j**) show the data processing steps for the fish fin and fish eye, respectively.

The applicability of this approach to increase imaging speed is investigated on the basis of the results for a fish fin and a fish eye, as shown in Figure 6.

The position differences of the reference mirrors between the two channels in the experiments on the fish fin and fish eye are 1.25 mm and 1.88 mm, respectively. The dispersion compensation coefficients are $a_{2}=9650\times 10^{-30},\;a_{3}=778\times 10^{-45}$ in the experiment on the fin, and $a_{2}=10660\times 10^{-30},\;a_{3}=428\times 10^{-45}$ in the experiment on the fish eye. Figure 6a–d show the results of the fish fin, while Figure 6e–h are the results of the fish eye. Figure 6a,d,e,h are obtained using complete spectral data, and the results in Figure 6b,c,f,g are obtained using 50% spectral data. All images in Figure 6 are the reconstructed structural images that have been compensated for dispersion. Figure 6ig show the data processing steps for the fish fin and fish eye, respectively. The curves in Figure 6i are signals in the depth direction at the x = −0.44 mm position of the fish fin, and the curves in Figure 6g are signals at the x = −0.66 mm position of the fish eye. The blue, red, and black lines are signals that are not compensated for dispersion, compensated for dispersion, and the final reconstructed signals, respectively. Figure 6c,d,g,h are images obtained by stitching the images of the two channels after using the TCDE method to reconstruct the structural signal. In the figures, the green dotted frame indicates the structure of channel 1, the blue dotted frame indicates the structure of channel 2, and the settings of the channel 1 and the channel 2 are different in the two experiments.

We can see that the quality of the image obtained using 50% spectral data and CS method are similar to that obtained using the complete spectral data. Therefore, it can be concluded that the method proposed in this paper can increase the imaging speed without reducing the imaging depth when the spectral resolution of spectrometer is certain.

**Figure 7:**
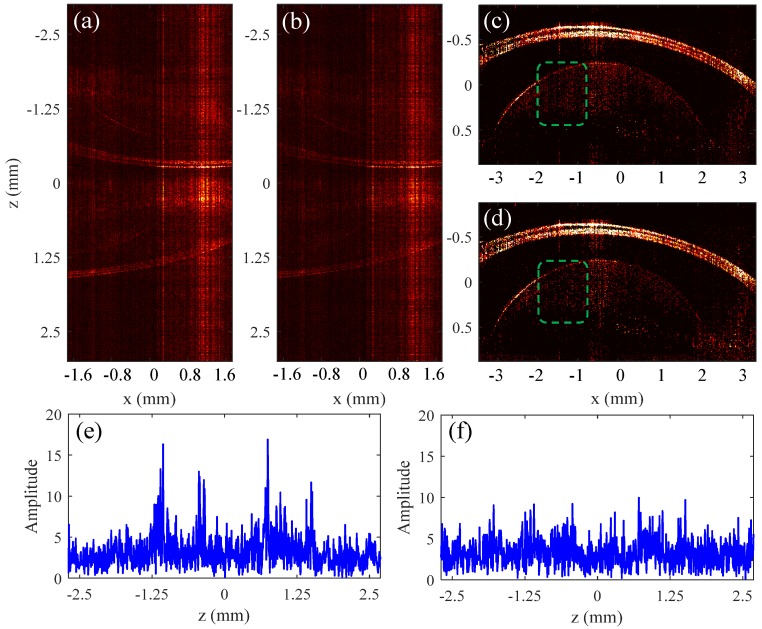
Experiment results of a fish eye. The results in (**a**,**c**,**e**) are obtained using 35% spectral data, and the results in (**b**,**d**,**f**) are obtained using 25% spectral data.

The results reconstructed from undersampled data with a sampling rate lower than 50% are summarized in Figure 7. Figure 7a,b show the dual-channel structure signals obtained from 35% data and 25% data, respectively. Figure 7c,d are reconstructed through the TCDE method from Figure 7a,b, respectively. In both cases, the corneal signals of fish eye are reconstructed well because they are larger. In contrast, compared to Figure 7c, many of the small signals in Figure 7d, especially those within the range shown by the green dashed box in Figure 7d, are not recovered due to the low sampling rate. In the process of reconstructing the structural image by the TCDE method, the residual signals after removing the large corneal signals at the position of *x* = 0.35 mm are shown in Figure 7e (35%) and Figure 7f (25%). It is obvious that the small signals in Figure 7e can be further recovered, but not in Figure 7f.

Therefore, the proposed method can reconstruct the fish-eye structure image from more than 35% of the data. The results of Figure 5 and Figure 7 demonstrate that the minimum sampling rate required to reconstruct the structure image actually depends on the magnitude of the backscatter signal and the sensitivity of the system.

**Figure 8:**
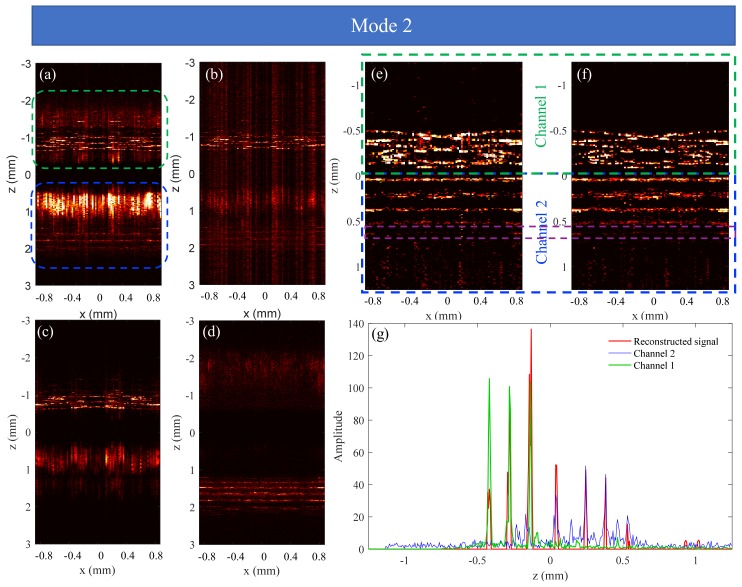
Experiment results of an onion. The results in (**a**,**c**), and (**d**) are obtained using complete spectral data, and the result in (**b**) is obtained using 50% spectral data. Panels (**e**) (50% data) and (**f**) (100% data) show images of an onion that are stitched together by the results of the two channels; Panel (**g**) shows the depth signals at *x* = 0 mm.

The applicability of this approach to extend imaging depth is investigated on the basis of the results for an onion, as shown in Figure 8. In the experiment, the second scanning scheme shown in Figure 2f-2 was used, and the structural images in Figure 8 were all corrected for the lateral position. That is, the deeper portion of the structural image was shifted to the right by an *x* distance, then the common portions of the upper and deeper structures were shown in Figure 8.

The position difference of reference mirrors between the two channels is 3.03 mm. The dispersion compensation coefficients are obtained: $a_{2}=9377\times 10^{-30},a_{3}=298\times 10^{-45}$. Figure 8c,d,a are structural images obtained by performing inverse Fourier transform on the complete spectral data of the channel 1, channel 2, and dual channels, respectively. The result in Figure 8b is obtained using 50% spectral data. The final stitched images using 100% data and 50% data are shown in Figure 8f,e, respectively. In Figure 8e, in order to suppress the noise of the image, we set a larger intensity threshold during the iterative process [23], thereby losing some structural information. For example, the structure signals in the purple dotted box of Figure 8f are erroneously removed in Figure 8e. The green, blue, and red curves in Figure 8g are the depth signals for channel 1, channel 2, and dual channels at x = 0 mm, respectively, from which we can see that the imaging depth is approximately doubled. Consequently, it can be concluded that the dual-channel SD-OCT system can extend the imaging depth without increasing imaging time and with lower spectral resolution of spectrometer compared to the single-channel SD-OCT system.

In this paper, the sample rate used in CS method is 50%. In fact, 50% of the spectral data is not enough to achieve the goal of halving the resolution of the spectrometer, since random sampling is required in CS. Nevertheless, this can be achieved with a lower sampling rate, such as 30%, and it has been demonstrated that approximately 30% of the spectral data can be used to reconstruct a high-quality OCT image [24]. For example, a spectrometer with a spectral resolution of 0.05 nm is required to measure the depth of *L*. We can undersample the data of the spectrometer with a spectral resolution of 0.025 nm at 70% sampling rate, and then reconstruct the image with depth of *L* using CS reconstruction algorithm, which is equivalent to reconstructing the image using 35% of the spectral data.

In this study, we only verified the feasibility of the dual-channel SD-OCT system, and tissues used in experiment are relatively simple. In the future, we will explore the imaging depth and imaging speed of this system when imaging a more complex biological tissue.

## 4. Conclusions

In conclusion, we design a dual-channel SD-OCT system based on a single spectrometer in this work. The two-channel data are separated by introducing an optical path offset between two channels in advance, and compressive sensing technology is used to avoid the decrease in imaging depth caused by this offset. We demonstrate the application feasibility of the dual-channel SD-OCT system combined with CS method in increasing imaging speed and expanding imaging depth by imaging a mirror, a fish fin, a fish eye, and an onion.

## Figures and Tables

**Table 1:** The parameters of the dual-channel SD-OCT system.

System Parameters	Specification
Lateral resolution (mode 1)	7.54 μm
Lateral resolution (mode 2)	3.77 μm
Depth of focus (mode 1)	452.16 μm
Depth of focus (mode 2)	113.04 μm
Axial resolution (air/tissue)	6.88 μm/ 4.74 μm
Maximum imaging depth (air/tissue)	3.12 × 2 mm/2.15 × 2 mm
Maximum imaging width	21.8 mm
